# A Comparative Study of *Lactarius* Mushrooms: Chemical Characterization, Antibacterial, Antibiofilm, Antioxidant and Cytotoxic Activity

**DOI:** 10.3390/jof9010070

**Published:** 2023-01-03

**Authors:** Marina Kostić, Marija Ivanov, Ângela Fernandes, Ricardo C. Calhelha, Jasmina Glamočlija, Lillian Barros, Marina Soković, Ana Ćirić

**Affiliations:** 1 Department of Plant Physiology, Institute for Biological Research “Siniša Stanković”—National Institute of Republic of Serbia, University of Belgrade, Bulevar Despota Stefana 142, 11000 Belgrade, Serbia; 2 Centro de Investigação de Montanha (CIMO), Instituto Politécnico de Bragança, Campus de Santa Apolónia, 5300-253 Bragança, Portugal; 3 Laboratório Associado para a Sustentabilidade e Tecnologia em Regiões de Montanha (SusTEC), Instituto Politécnico de Bragança, 5300-253 Bragança, Portugal

**Keywords:** *Lactarius vellereus*, *Lactarius quietus*, *Lactarius piperatus*, biofilm

## Abstract

Mushrooms are valued worldwide for their nutritional, organoleptic and chemical properties. The aim of this study was to determine the chemical composition (free sugars, organic acids, fatty acids, tocopherols and phenolic compounds) and bioactivity of three wild mushrooms (*Lactarius piperatus*, *Lactarius quietus* and *Lactarius vellereus*) from Serbia. Chemical analysis was performed with HPLC-RI and UFLC-PDA (for hydrophilic compounds) and with GC-FID and HPLC-FP (for lipophilic compounds). The analysis of phenolic compounds was performed by UFLC-DAD. Biological activities were evaluated using three different assays (microdilution, TBARS and SRB assays). The results showed that the fruiting bodies were rich in mannitol and trehalose. The main organic acids were oxalic acid and citric acid. As for lipophilic components, stearic, oleic and linoleic acids and β-tocopherol dominated in all the species studied. In addition, the methanolic and ethanolic extracts obtained showed antibacterial, antibiofilm and antioxidant properties. As for cytotoxicity, the extracts were not toxic or only moderately toxic toward different tumor cell lines. According to the results, the selected Serbian mushrooms are a rich source of bioactive compounds, and due to their good biological potential, they can be further exploited as functional ingredients beneficial to human health (antimicrobial agents, antioxidants).

## 1. Introduction

Many mushrooms (edible and/or medicinal) represent an underutilized source of substances that can be used in the treatment of various human diseases. Due to their high content of proteins and secondary metabolites, mushrooms are primarily used to improve the immune response to various diseases. Nowadays, different dietary products based on mushrooms are used for the prevention as well as alleviation of the symptoms of different diseases. Mushroom-based products cannot completely replace commercial drugs, but in combination with their use, they can lead to an improvement in the general condition of the patient [1,2,3]. Considering factors such as nutrition, health care and socioeconomic changes to supplement existing food and drugs, the cultivation and commercialization of wild-growing mushrooms that can serve as a nutraceutical, foods, and/or pharmaceutical ingredient is a promising area of research for scientists [1]. Native species are therefore an important and unexplored source of biologically active compounds that may have applications in medicine, pharmacy and various areas of the food industry.

The genus *Lactarius* Pers. (Russulales) is one of the largest genera within the Basidiomycotina; over 600 species of the genus have been described worldwide [4]. They are found in deciduous forests, and the characteristic by which these mushrooms are recognized is a milky fluid that they exude when their basidiocarp is broken. Because of the production of the milky liquid, the entire genus is referred to as “milk cap”. *Lactarius* species are ectomycorrhizal species, meaning that they play an ecological role in terrestrial ecosystems through relationships with various plants [4]. In addition, many *Lactarius* species are considered medicinal, nutritionally important and promising mushrooms [5,6,7]. There are some studies on *Lactarius piperatus* (L.) Pers. and *Lactarius vellereus* (Fr.) Fr. reporting their mineral [8,9] and chemical composition (nutritional value, protein, fatty acids, tocopherols and phenolic acids contents) [5,10,11,12]. Also, researchers have investigated the antimicrobial activity against different ATCC strains [5,9,13,14,15] and antioxidant activity (including DPPH scavenging activity, reducing power, β-carotene bleaching inhibition, TBARS inhibition activity) [5,9,10,13,15] of these species. Ding et al. [7] have identified LV-1 and CM-S (new polysaccharides from *L. vellereus*) and determined their immunoactivity. Literature data regarding *L. quietus* (Fr.) Fr. chemical composition and bioactivity are scarce [16]. The mentioned studies refer to the species that grow in different European countries (Portugal, Turkey and Romania). Nevertheless, there are no detailed studies on the chemical profile of the abovementioned Serbian *Lactarius* species. Few papers have been published on Serbian *Lactarius* wild-growing mushrooms such as *L. deliciosus*, *L. volemus*, *L. sanguifluus*, *L. semisanguifluus* and *L. piperatus* [17,18]. The focus of these studies was to investigate their biological properties (antioxidant, antimicrobial, cytotoxicity, geno- and neuro-protective activity) and the determination of total phenols. Therefore, none of the previous studies provided a detailed chemical analysis and explained the correlation between the detected chemical compounds with various biological activities.

The aim of our study was to increase the knowledge on the *Lactarius* species from Serbia by presenting a detailed chemical characterization of *L. piperatus, L. vellereus* and *L. quietus*, including an evaluation of the hydrophilic (free sugars and organic acids), lipophilic (fatty acids and tocopherols) and phenolic compounds, as well as the biological activities of their methanolic and ethanolic extracts. The evaluation of biological activity included the examination of antibacterial and antibiofilm potential against oral pathogens, the determination of antioxidant activity (inhibition of lipid peroxidation) and the evaluation of cytotoxicity (against non-tumor and tumor cell lines).

## 2. Materials and Methods

### 2.1. Collection of Lactarius Species

For chemical characterization and analysis of the biological activity of the extracts, the fruiting bodies of three *Lactarius* species (Table 1) were collected from different locations in the Republic of Serbia. Identification of the mushrooms was based on macroscopic and microscopic characteristics of fruiting bodies, by comparison with available keys for the identification of fungi [19]. Fresh fruiting bodies were brought to the laboratory on the same day as the harvest. The mushrooms were then thoroughly cleaned, weighed (1 kg per species) and frozen in polyethylene bags at −20 °C for 24 h before lyophilization. Lyophilization (LH Leybold, Lyovac GT2, Frankendorf, Switzerland) of each sample took 10 days. After 10 days, each sample was ground to a fine powder and stored at 4 °C in hermetically sealed high-density polyethylene bags for further analysis (which was performed a few days after lyophilization). The fruiting bodies of the *Lactarius piperatus* (L.) Pers., peppery milk cap mushroom, were obtained in September 2017, in the areas of the mountain Kosmaj south of Belgrade, under live beech trees (*Fagus sylvatica*) and oak trees (*Quercus cerris*). Basidiocarps of *Lactarius quietus* (Fr.) Fr., oak milkcap, were collected in Divčibare on the slopes of the Maljen mountain at an altitude of 1000 m in oak forests during the autumn months of 2014. Mushroom samples of *Lactarius vellereus* (Fr.) Fr., fleecy milk-cap, were collected in October 2016 in mixed stands of deciduous and coniferous forests on Kopaonik mountain (central Serbia).

### 2.2. Extracts Preparation

Methanolic (ME) and ethanolic (EE) extracts of the fruiting bodies of the selected species were used to test the biological activities. Extracts were prepared according to the procedures described by Kostić et al. [20] and Fernandes et al. [21].

### 2.3. Chemical Analysis of the Basidiocarps of the Tested Mushrooms

#### 2.3.1. Hydrophilic Compounds

Free sugars were determined by a high-performance liquid chromatography (HPLC) system (Knauer, Smartline system 1000, Berlin, Germany) coupled with a refraction index detector (RI detector, Knauer Smartline 2300). The extraction and whole procedure were performed according to Barros et al. [22]. Compound identification was made by comparing the relative retention time of sample peaks with internal standard (IS, raffinose). The results were expressed in g per 100 g of dw.

Organic acids were determined by ultra-fast liquid chromatography coupled with a photodiode array detector (PDA), (UFLC, Shimadzu 20A series, Kyoto, Japan) following a procedure described by Barros et al. [23]. The quantifications were calculated by comparing the area of their peaks, recorded at 215 nm, with the calibration curves obtained from the commercial standards, and results were expressed in g per 100 g of dw.

#### 2.3.2. Lipophilic Compounds

Fatty acids were determined using a gas chromatographer (DANI 1000) equipped with a split/splitless injector and a flame ionization detector (GC-FID) [22]. The identification of the fatty acids was made by comparing the relative retention times of FAME peaks from samples with standards. Results were recorded and expressed as the relative percentage of each fatty acid.

Following the procedure described by Heleno et al. [24], tocopherols were extracted and determined by HPLC with a fluorescence detector (FP-2020; Jasco, Easton, MD, USA). Compounds were identified by chromatographic comparison with commercial standards and quantification was based on the fluorescence signal response of each standard, using the IS (tocol) method and calibration curves obtained from the commercial standards of each compound. The tocopherol content was expressed in μg per 100 g of dw.

#### 2.3.3. Phenolic Compounds

Phenolic compounds were analyzed with UFLC equipment coupled to a diode array detector (DAD) [21]. The phenolic acids and related compounds were quantified by comparison of the area of their peaks, recorded at 280 nm, with calibration curves obtained from commercial standards. The results were expressed in μg per g of extracts.

### 2.4. Biological Activity of Lactarius Species

The following methods were used to test the biological activity of mushroom extracts in vitro:

#### 2.4.1. Antibacterial Potential

Testing of the antibacterial activity of selected mushroom extracts was done using the microdilution method [20,25]. The following oral isolates of Gram-positive (*Micrococcus luteus, Rothia mucilagenosa, Streptococcus agalactiae, Streptococcus angiosus, Streptococcus conselatus, Streptococcus dysgalactiae, Streptococcus oralis, Streptococcus parasanquinis, Streptococcus pseudopneumoniae, Streptococcus pyogenes, Streptococcus salivarius, Staphylococcus aureus, Staphylococcus hominis, Staphylococcus warneri*) and Gram-negative (*Stenotrophomonas maltophilia* and *Enterobacter cloacae*) bacteria were used. They were obtained from the tonsillar tissue of patients, at the Otorhinolaryngology clinic at Clinical Hospital Center Zvezdara, Belgrade, Serbia [26]. Tested bacteria were deposited at the Mycological Laboratory, Department of Plant Physiology, Institute for Biological Research “Siniša Stanković”, National Institute of the Republic of Serbia, University of Belgrade. Minimum inhibitory (MIC) and minimum bactericidal concentrations (MBC) were determined. Amoxicillin with clavulanic acid (Hemofarm, Vršac, Serbia) and cefixime (Alkaloid, Skoplje, Macedonia) were used as positive controls.

#### 2.4.2. Antibiofilm Potential

Inhibition of *Staphylococcus aureus* Biofilm Formation

*S. aureus* samples were incubated with MIC and sub-MIC of the examined agents in Tryptic soy broth with 2% glucose at 37 °C for 24 h. After incubation, the plate was washed twice with sterile PBS and fixed with methanol for 10 min. Methanol was removed by pipetting and the plate was air-dried. The biofilms were stained by using 0.1% crystal violet (Sigma Aldrich, Darmstadt, Germany) for 30 min, water washed and air dried, and the stain was dissolved in 96% ethanol (Zorka, Sabac, Serbia). The absorbance was read at 570 nm on a Multiskan™ FC microplate photometer (Thermo Scientific™, Waltham, MA, USA) and the results are presented as the inhibition of biofilm formation (%) [27].

Inhibition of the formed *Staphylococcus aureus* biofilm

The *S. aureus* inoculum was incubated in a microtiter plate with an adhesive bottom for 24 h at 37 °C. After incubation, the plate was washed 3× with saline and the adhered cells were treated with MBC of extracts for 30 s. After washing, addition of methanol, drying, staining with crystal violet dye and addition of ethanol, the absorbance was read at a wavelength of 570 nm on an automated Elisa reader (Multiskan™ FC Microplate Photometer, Thermo Scientific), and the percentage of biofilm diminishing was calculated [28].

#### 2.4.3. Antioxidant Activity Measured by Thiobarbituric Acid Reactive Substances Assay—TBARS

Detection of lipid peroxidation using the TBARS method was performed according to the method of Reis et al. [16]. For the purposes of the experiment, fresh pig brain (*Sus scrofa*) was used. The brain was homogenized and the homogenate was centrifuged at 3000 g for 10 min, then aliquots of the supernatant (100 μL) were incubated for 60 min with 200 μL of extracts of different concentrations in the presence of ascorbic acid (0.1 mM; 100 μL) and FeSO_4_ (10 mM; 100 μL) at 37 °C. The reaction was stopped by adding 500 μL of trichloroacetic acid (28% *w*/*v*, 380 μL) and then thiobarbituric acid (2% TBA, 0.38 mL). Then, the mixture was heated for 20 min at 80 °C and centrifuged for 5 min at 14,000 rpm to remove the precipitated proteins. The supernatant contained a pink MDA-TBA complex with an absorption maximum at 532 nm. The results were calculated and expressed in mg/mL.

#### 2.4.4. Cytotoxicity Evaluation

The cytotoxicity was evaluated in human tumor cell lines (MCF-7—breast adenocarcinoma, NCI-H460—non-small-cell lung cancer, HCT-15—colon carcinoma, HeLa—cervical carcinoma and HepG2—hepatocellular carcinoma) and in a non-tumor liver cell primary culture (PLP2). The methanolic and ethanolic extracts of three different *Lactarius* species were dissolved in water (8 mg/mL) and ellipticine was used as the positive control. All results were expressed as the sample concentration that inhibited 50% of the net cell growth (GI_50_ values, μg/mL) [29].

### 2.5. Statistical Analysis

The experiments were performed in three repetitions and the results were expressed as arithmetic mean value ± standard error of measurement. Results were analyzed using one-way and two-way ANOVA, Tukey’s HSD test with *p* < 0.05 and Student’s *t*-test with *p* < 0.05. Statistical analyzes were performed using SPSS Statistics software (IBM SPSS 148 Statistics for Windows, Version 22.0. Armonk, NY, USA: IBM Corp.) and GraphPad Prism 6 software.

## 3. Results

### 3.1. Chemical Composition of Lactarius Species

The results of the chemical analysis of sugar content in basidiocarps of the studied species showed the presence of trehalose and mannitol (Table 2). Among the tested species, no fructose was detected in the basidiocarps of the *Lactarius* species. The range of values of mannitol content was from 3.67 to 13.47 g/100 g dw. The highest value was measured in the basidiocarps of *L. vellereus*, with 13.47 g/100 g dw, while the lowest value of mannitol was present in the basidiocarps of *L. quietus* (3.67 g/100 g dw). The trehalose content of the analyzed species was as follows (ascending order): *L. quietus* (0.34 g/100 g) < *L. vellereus* (0.77 g/100 g) < *L. piperatus* (17.78 g/100 g). The obtained results showed that the total content of identified sugars of the studied mushrooms varied in a wide range from 4.01 g/100 g dw (*L. vellereus*) to 23.59 g/100 g dw (*L. piperatus*).

The results regarding the organic acids for the wild-growing *Lactarius* mushroom species are shown in Table 2. The range of oxalic acid values was from 1.9 (*L. piperatus*) to 3.9 g/100 g dw (*L. vellereus*). Quinic acid and malic acid were detected only in *L. piperatus* (1.9 g/100 g dw; 6 g/100 g dw, respectively). Malic acid was detected only in *L. piperatus* (6 g/100 g dw). Citric acid was found in small amounts in the basidiocarps of *L. piperatus* (0.5 g/100 g dw), while it was detected in large amounts in *L. vellereus* (8.5 g/100 g dw). Fumaric acid was also determined in small amounts in *L. piperatus*, while it was found in trace amounts in *L. quietus* and *L. vellereus*. Shikimic acid was quantified only in basidiocarps of *L. vellereus* (0.49 g/100 g dw). The obtained results show that the total content of identified organic acids of the tested mushrooms varies in a wide range from 3.7 g/100 g dw in *L. quietus* to 12.4 g/100 g dw in *L. vellereus*.

The results of the fatty acids analysis are presented in Table 3. Palmitic acid (C16:0), stearic acid (C18:0), oleic acid (C18:1n9c), and linoleic acid (C18:2n6c) were the most abundant fatty acids in all species analyzed (Table 3). However, different fatty acids were dominant in all three species. Stearic acid (42.1%) and oleic acid (36.9%) dominated in the basidiocarps of *L. piperatus*, while linoleic acid (11.9%) and palmitic acid (4.93%) were much less abundant. In *L. quietus*, the content of oleic acid (40.4%) and linoleic acid (19.2%) was similar to that of *L. piperatus*, whereas the content of palmitic acid (20.1%) and stearic acid (10.1%) was different. Stearic acid (58.2%) was the dominant fatty acid in the basidiocarp of *L. vellereus*, while oleic acid (17.6%), linoleic acid (12.15%) and palmitic acid (10.0%) were significantly less present. In addition to those mentioned, other fatty acids are represented with a proportion of below 2% or were not detected at all in some basidiocarps. The analysis of the ratio of the total content of saturated, unsaturated and polyunsaturated fatty acids showed that the ratios varied from species to species. Thus, in *L. piperatus* and *L. vellereus*; saturated (SFA) dominated, whereas, in *L. quietus*, unsaturated fatty acids (MUFA) were the most abundant (Table 3).

The presence and content of four tocopherol isoforms were analyzed: α, β, γ and δ; the results are shown in Table 3. α-tocopherol was detected only in *L. piperatus* (4 μg/100 g dw). This is in contrast to β-tocopherol, which was detected in all three tested *Lactarius* species. The range of β-tocopherol content was 47 μg/100 g dw (*L. piperatus*)—1391 μg/100 g dw (*L. vellereus*). The γ- and δ-tocopherols were found only in the basidiocarps of *L. piperatus* (26.1 μg/100 g dw and 3.1 μg/100 g dw, respectively). The presence of all tocopherol isoforms was detected only in the *L. piperatus*, with β-tocopherol being the dominant isoform. The obtained results showed that the total content of the identified tocopherol isoforms of the studied mushrooms ranged from 69.0 μg/100 g of dw (*L. quietus*) to 1319 μg/100 g of dw (*L. vellereus*).

The results of phenolic acid content in the methanol and ethanol extracts of the studied *Lactarius* species are presented in Table 4. *p*-Hydroxybenzoic acid, cinnamic acid and protocatechuic acid were identified (Table 4). *p*-Hydroxybenzoic acid was identified in both extract types for *L. quietus* and *L. vellereus* species. For the aforementioned species, the highest levels of *p*-hydroxybenzoic acid were detected in the samples of methanolic (795 μg/g ext) and ethanolic (4735 μg/g ext) extracts of *L. quietus*. Cinnamic acid was identified in ME species *L. piperatus* (8.5 μg/g ext)*, L. quietus* (23.8 μg/g ext) and in EE of the same species (5.2 μg/g ext and 126 μg/g ext, respectively). Protocatechuic acid was found only in the species *L. vellereus* in ME (4.2 μg/g ext) and EE (11.4 μg/g ext). The EE of *L. quietus* had the highest content of total phenols.

### 3.2. Biological Activities of Lactarius Species

Antibacterial activity was determined by the microdilution method and the degree of sensitivity of 16 tested bacteria to methanolic (ME) and ethanolic extracts (EE) of *Lactarius* species was shown in Table 5. Depending on *Lactarius* species and bacterial type, the MICs ranged from 0.20 to 25 mg/mL. The bacteria that were most sensitive to *Lactarius* ME were: *Streptococcus agalactiae* and *Streptococcus parasanqinis* (MIC 0.20–0.80 mg/mL, MBC 0.8–1.6 mg/mL), while the most resistant were *Staphylococcus aureus* (MIC 12.5–30 mg/mL, MBC 25–30 mg/mL) and *Stenotrophomonas maltophilia* (MIC 6.25–25 mg/mL, MBC 12.5–50 mg/mL). The inhibitory activity of EE varied from 0.20 to 25 mg/mL (Table 5). The most sensitive bacteria were: *Rothia mucilaginosa*, *S. agalactiae*, *S. anginosus*, *S. dysgalactiae*, *S. parasanqinis* and *S. pyogenes* (MIC 0.60–0.80 mg/ mL), while *S. aureus* (MIC 12.5–25 mg/mL, MBC 25–50 mg/mL) and *S. maltophilia* (MIC 12.5–25 mg/mL, MBK 25–50 mg/mL) were the most resistant to ethanolic extracts. Commercial antibiotics amoxicillin with clavulanic acid and cefixime, whose MIC values ranged from 0.0002–0.028 mg/mL (MBC 0.0004–0.056 mg/mL) and 0.0002–0.013 mg/mL (MBC 0.0004–0.027 mg/mL), respectively, showed better antibacterial activity against all tested bacteria than the tested extracts. Comparing the antibacterial activity of ME among all three *Lactarius* species, there were no significant differences. All methanolic extracts of *Lactarius* showed consistent activity. Uniform antibacterial activity was also observed in all EE *Lactarius* species tested. However, a comparative analysis of the bacteriostatic and bactericidal effects of ME and EE showed that ME has slightly better activity compared to EE.

**Figure 1:**
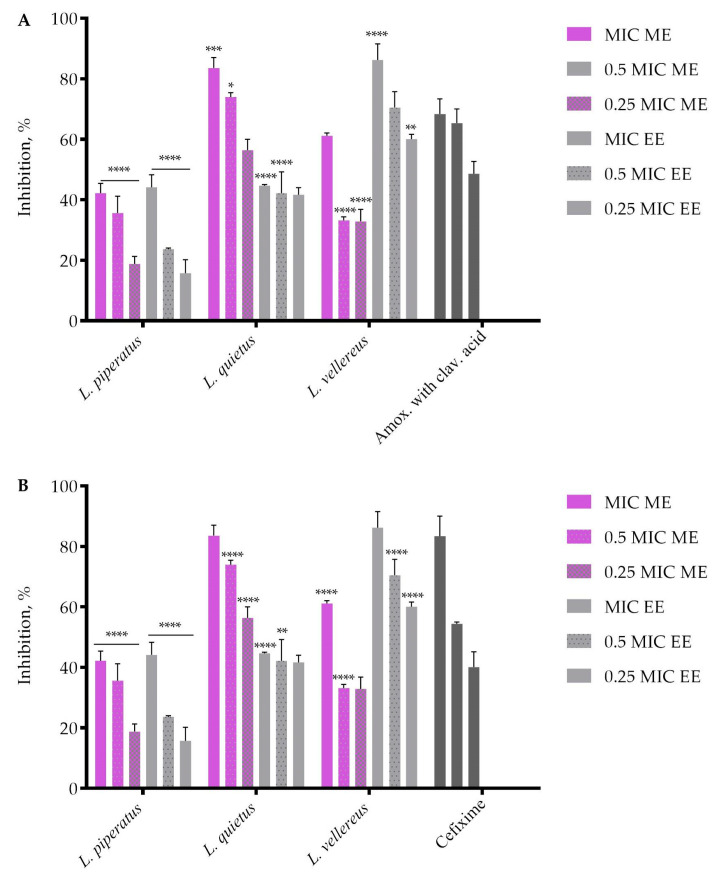
Formation of *S. aureus* biofilm after treatment with *Lactarius* extracts compared to commercial drugs: (**A**) amoxicillin with clavulanic acid; (**B**) cefixime. The error bars indicate standard deviations. The asterisks represent statistical significance *, *p* ≤ 0.05; **, *p* ≤ 0.01; ***, *p* ≤ 0.001, ****, *p* ≤ 0.0001, the data are presented as mean ± SD.

The concentrations of the extracts used to study the degree of inhibition of *S. aureus* biofilm formation were: MIC, 0.5 MIC and 0.25 MIC (Figure 1). The obtained results showed that the inhibition of biofilm formation depended on the concentration of the extract. The best effect on biofilm inhibition at all tested concentrations was shown by ME *L. quietus* (83.58%; 73.97% and 56.39%) and by *L. vellereus* EE (86.2%; 70.47%; 60.1%) (Figure 1). A comparative analysis showed that ME *L. quietus*, and EE *L. vellereus*, had a better effect on inhibiting biofilm formation than the tested amoxicillin with clavulanic acid (Figure 1A) and cefixime (Figure 1B).

**Figure 2:**
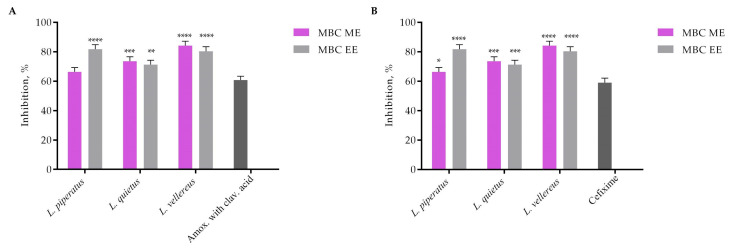
Destruction of *S. aureus* 24 h preformed biofilm after 30 s treatment with *Lactarius* extracts compared with commercial drugs: (**A**) amoxicillin with clavulanic acid; (**B**) cefixime. The error bars indicate standard deviations. The asterisks represent statistical significance *, *p* ≤ 0.05; **, *p* ≤ 0.01; ***, *p* ≤ 0.001, ****, *p* ≤ 0.0001, the data are presented as mean ± SD.

The effect of ME and EE of the tested mushrooms after treatment (30 s) with minimum bactericidal values (MBCs) on the formed *S. aureus* biofilm is shown in Figure 2. Comparing the results within the *Lactarius* species, the extracts of the *L. vellereus* species (ME—84.2% and EE—80.4%) had the best effect on the inhibition of formed biofilm (Figure 2).

All tested extracts showed a better effect on the formed biofilm compared to commercial antibiotics, whose inhibitory effect on MBC values was 61.14% for amoxicillin and clavulanic acid and 59.05% for cefixime. The comparative analysis showed that *L. piperatus* EE and *L. vellereus* ME and EE (*p* ≤ 0.0001) had a better effect on the inhibition of the formed biofilm than the tested antibiotics (Figure 2A,B).

The antioxidant activity of the extracts was evaluated by their ability to inhibit lipid peroxidation (TBARS test, Table 6). *L. quietus* ME and EE showed the best activity (IC_50_ value for ME was 0.17 mg/mL, i.e., IC_50_ EE 0.23 mg/mL), while the lowest activity was found for *L. piperatus* EE (IC_50_ 1.39 mg/mL). Considering that organic and phenolic acids are responsible for the antioxidant activity of mushroom extracts, the results of the chemical characterization of the tested species (Table 2 and Table 4) are consistent with the TBARS values of the tested *Lactarius* species. Based on the potential to inhibit lipid peroxidation in the ME of the tested species in ascending order was as follows: *L. quietus* < *L. vellereus* < *L. piperatus*, with the lowest value indicating the best activity. While the EE effect of the tested mushrooms in ascending order was: *L. quietus* < *L. vellereus* < *L. piperatus*. Based on the obtained results, the ME showed better antioxidant activity detected by TBARS tests than the EE from all tested species.

The effects of six different mushroom extracts on the growth of porcine liver primary culture (PLP2) and four human tumor cell lines (HeLa, HepG2, MCF-7 and NCI-H460) were determined, and the GI_50_ values are detailed in Table 6. Most of the tested *Lactarius* extracts had no cytotoxic effects toward non-tumor liver primary cells at the tested concentrations (PLP2; GI_50_ > 400 μg/mL; Table 6). Only *L. quietus* ME and *L. vellereus* EE showed some reduction in cell growth with GI_50_ 347 μg/mL and 163 μg/mL, respectively. The ME of *L. piperatus* showed activity against all tumor cell lines, except for NCI-H460, whereas the EE was cytotoxic only against HeLa and MCF-7 tumor cell lines. The ME of *L. quietus* expressed activity against all tested tumor cell lines, while *L. quietus* EE demonstrated cytotoxic potential only against HeLa. The ME of *L. vellereus* showed activity against the HeLa, HepG2 and NCI-H460 cell lines. Of all tested extracts, the *L. vellereus* EE showed the most promising cytotoxic activity.

## 4. Discussion

Researchers have shown that the free sugar content in the fruiting bodies of wild species may vary due to their different origins. The ranges of values of trehalose content in different stages of maturity of the fruiting body of *L. piperatus* from Portugal was 0.83–1.20 g/100 g [30]. Trehalose was also the predominant sugar in the sample from Serbia, but in a much larger amount (17.8 g/100 g dw). Alcoholic sugars, especially mannitol, are responsible for the firmness and volume of the fruiting body of mushrooms, which explains the higher content of mannitol in the more mature fruiting bodies of *L. piperatus* [5]. Reis et al. [16] reported that basidiocarps of *L. quietus* species contain 9.84 g/100 g dw mannitol and 4.73 g/100 g dw trehalose, while the values of the mentioned sugars were significantly lower in the studied sample from Serbia (Table 2). In contrast to the sugar content of the species *L. piperatus* and *L. quietus*, the basidiocarps of *L. vellereus* originating from Portugal were richer in mannitol (24.77 g/100 g of dw), while trehalose was present in a lower amount (2.41 g/100 g of dw) [10]. The total sugar content in the fruiting bodies of samples from Portugal is in descending order: *L. vellereus* > *L. quietus* > *L. piperatus*; while the total sugar content in the fruiting bodies of the tested samples from Serbia was as follows: *L. piperatus* > *L. vellereus* > *L. quietus*. Qualitative and quantitative analysis of sugars is important for several reasons. First, because they affect the taste of edible mushrooms (by enhancing the sensation of sweet taste) and because the amount of sugar is an important indicator of storage and preservation conditions [31,32]. In addition, mannitol, the most abundant sugar in most mushrooms, plays an important role in regulating osmotic pressure in cells and removing RONS [33]. For example, Zhao et al. [33] showed that under stress conditions such as low temperature, the synthesis of mannitol was inhibited at the initial stage of *Volvariella volvacea* growth. They also found that the application of mannitol solution at the cultivation phase of *V. volvacea* improved the resistance of fruiting bodies to low temperatures, confirming the correlation between mannitol and resistance to this type of stress.

For the species examined in this study, no information was found on organic acid content. However, there are data on the profile of organic acids in other species of the genus *Lactarius*. Vieira et al. [6] identified and quantified oxalic acid, malic acid, fumaric acid and quinic acid in samples of basidiocarps of *Lactarius turpis* and *Lactarius citrolens*, with oxalic acid dominating in the sample of *L. turpis* and malic acid and fumaric acid in the sample of *L. citrolens*. The same authors state that quinic acid was detected only in the sample of *L. citrolens*. The content of organic acids was also studied in the *Lactarius volemus* and *Lactarius deliciosus* species originating from Portugal, and the studies showed that malic acid dominated in both samples [23,34]. Studies have shown that organic acids have diverse biological potential. Thanks to their ability to chelate metals, organic acids such as malic and citric acid act as antioxidants. Citric acid also has antibacterial properties and is reported as a very important compound in preventing mushroom browning [23,35]. Oxalic acid and fumaric acid are known as antimicrobial and antitumor agents. Fumaric acid, in addition to its antimicrobial and antitumor effects, has anti-inflammatory, neuroprotective and chemopreventive potential [35].

The high content of unsaturated fatty acids in mushrooms is very important from the point of view of human health. The most abundant fatty acids in the basidiocarps of *L. piperatus* from Portugal was stearic acid, followed by linoleic and oleic acids [30]. The total SFA content was also higher than that of unsaturated fatty acids, which is in accordance with the values obtained in this study. In the Romanian species, linoleic acid dominated, while PUFA were the most abundant, which is contrary to the results of this study [12]. The dominant fatty acids in the basidiocarps of *L. quietus* from Portugal were linoleic acid (35.15%) and oleic acid (24.55%) [16], as well as in the tested sample from Serbia (19.2%, i.e., 0, 40%). The sample from Portugal is richer in PUFA, while the basidiocarp sample of *L. quietus* from Serbia contains more MUFA. According to Heleno et al. [10], the dominant fatty acids in the basidiocarps of *L. vellereus* were stearic (58.33%) and linoleic acids (22.13%), also the SFA content in the sample from Portugal is significantly higher than the MUFA and PUFA content. The fatty acid content of the *L. vellereus* sample from Portugal is in agreement with the results obtained for the basidiocarps of *L. vellereus* from Serbia (Table 3). Fatty acids are important due to their anti-inflammatory, hypolipidemic and vasodilatory properties, and preventive effects on cardiovascular diseases and hypertension [36]. Su et al. [37], showed that unsaturated fatty acids can delay the breakdown of carbohydrates in the intestine and prevent a rise in glucose levels after a meal. Linoleic and oleic acids are two of the most important fatty acids. Linoleic acid is necessary for optimal functioning and fat balance in the human body [38]. Cozge et al. [39], showed that the dominant fatty acid in the seeds of different varieties of safflower oil was linoleic (54.2–81.5%) followed by oleic acid (9.98–36.63%), while other studies demonstrated that the content of oleic acid in sesame and sunflower oils ranges from 32.5 to 36.3% [40,41]. The percentage of oleic acid in the different plant oils is consistent with the content of this specific fatty acid in tested mushrooms (Table 3). Given that oleic acid (monounsaturated fatty acid) is responsible for many biological activities, including cardio-protective effects, regulation of blood pressure, insulin and glucose levels, mushrooms can be considered a valuable source of fatty acids important for human health according to the results.

Mushroom fruiting bodies contain tocopherols in significant amounts. No data were found in the literature about the tocopherol content of *L. piperatus*. According to Reis et al. [16], their sample of *L. quietus* contains three isoforms of tocopherols (α-, γ- and δ- tocopherol), of which the dominant isoform was α-tocopherol (47.98 μg/100 g of dw), in contrast to the sample from Serbia, in which only β-tocopherol (69.0 μg/100 g dw) was detected. However, in the sample of *L. vellereus* originating from Portugal, four isoforms of tocopherol were detected, with the dominant form being β-tocopherol (242.41 μg/100 g dw) [10], while the sample from Serbia was unusually rich in only β-tocopherols (1391 μg/100 g dw). According to data from the literature, the dominant isoforms within the genus *Lactarius* varied between the species, as well as the total tocopherol content, which varied from 15 μg/100 g to 316 μg/100 g [6,10,16,24]. The biological potential of tocopherols is reflected in their antioxidant potential, as these compounds interact with peroxyl radicals in membranes to form stable lipid hydroxyl peroxides [24]. Tocopherols also have a preventive effect on the formation of radicals in the membranes and tissues of biological systems, so they are considered to reduce the risk of cardiovascular and malignant diseases [24,42]. Bourgarrou et al. [43] reported that yogurt enriched with a tocopherol-rich extract of *Ganoderma lucidum* had better antioxidant activity than yogurt supplemented with commercial tocopherol. Therefore, it is very important to analyze the tocopherol content of various wild mushrooms, so that their extracts can be used for food fortification because in this way tocopherols from natural sources could replace commercial synthetic antioxidants and improve human nutrition and the quality of life.

Cinnamic, *p*-hydroxybenzoic, caffeic, *p*-coumaric, protocatechuic and gallic acids are the most abundant phenolic acids found in mushrooms [44] Barros et al. [45] quantified only *p*-hydroxybenzoic acid in the sample of *L. delicious* (22.66 mg/kg dw), while cinnamic acid (0.70 mg/100 g dw) was present in the sample of *L. quietus* sample from Portugal [16]. The extract of *L. vellereus* from Portugal showed the presence of protocatechuic (0.99 mg/100 g dw), *p*-hydroxybenzoic (0.16 mg/100 g dw), *p*-coumaric (0.18 mg/100 g dw) and cinnamic acids (1.07 mg/100 g dw) [10], while the presence of protocatechuic and *p*-hydroxybenzoic acids was also confirmed in the analyzed extracts from Serbia (Table 4). In both cases, protocatechuic acid was dominant. Regarding the profile of phenolic compounds of *L. piperatus*, Fogarasi et al. [12] detected 4-Feruloylquinic acid (87.62 g/fw), 3-Feruloylquinic acid (66.73 g fw) and 4-Hydroxybenzoic acid (42.93 g fw) as the most abundant in this Romanian wild mushroom. Many phenolic compounds are responsible for numerous biological activities, including anti-tumor, anti-inflammatory, antioxidant, antihyperglycemic, antiosteoporotic, anti-tyrosinase and antimicrobial activities [46,47,48]. *p*-Hydroxybenzoic acid, which was the most abundant in the *L. quietus* sample among the tested mushrooms is proven to possess anti-inflammatory activity. It also has a significant effect as a tyrosinase inhibitor [44]. Protocatechuic acid has been shown to have antioxidant [49], antimicrobial [48], cytotoxic and anti-inflammatory [50] as well as neuroprotective [51] effects, while cinnamic acid has pronounced antitumor effect on the NCI-H460 malignant cell line [52]. In addition to the above properties, phenolic acids also have UV protective activity as well as insecticidal activity [53].

According to the World Health Organization, bacterial infections caused by multidrug-resistant strains (e.g., *Staphylococcus aureus*, *S. epidermis*, *Streptococcus* sp., *Enterococcus* sp. and *Escherichia coli*) are increasing. As a result, the has been an increase in the number of studies focused on the analysis of various extracts and compounds isolated from mushroom fruiting bodies to find new compounds with antimicrobial activity [54]. The methanolic extract of *L. piperatus* from Portugal inhibited the growth of various types of pathogenic bacteria, including *S. aureus* (MIC 0.5–50 mg/mL, zone of inhibition 6–>9 mm) [5], while the acetone extract of *L. piperatus* from Serbia showed better inhibitory activity (MIC value range 0.039–0.156 mg/mL), including *S. aureus* (MIC 0.039 mg/mL) [17]. Likewise, methanolic extract from Romanian *L. piperatus* demonstrated similar activity against the tested *S. aureus* ATCC 49444 strain (MIC 26.99 mg/mL) [9]. The MIC values obtained for *L. piperatus* samples from Portugal and Romania are in agreement with the results obtained in this manuscript. Different extracts (methanolic, acetate and chloroform) of *L. vellereus* from Turkey inhibited the growth of G (+) and G (−) bacteria very well, MIC ranged from 0.0048 to 0.078 mg/mL. In the same study, the acetate extract of *L. vellereus* had the best activity on *B. subtilis* (MIC 0.0048 mg/mL) and *K. pneumoniae* (MIC 0.019 mg/mL). For growth inhibition of *S. aureus* and *S. pyogenes*, all three tested extracts showed the same activity (MIC 0.039 mg/mL) [13]. The antimicrobial activity values for the sample tested in this manuscript are higher than those of the tested sample from Turkey. The reasons for different MIC values may be: species origin, storage and extraction methodology. Alves et al. [48] confirmed that phenolic compounds were the most active compounds against different bacterial strains (*Staphylococcus aureus, S. epidermidis, Enterococcus faecalis, Listeria monocytogenes, Escherichia coli, Proteus mirabilis and Morganella morganii*). They identified 2,4-dihydroxybenzoic and protocatechuic acids as the major phenolics with high antimicrobial activity. Moreover, this study highlighted that carboxylic groups in their molecular structure play an important role in antimicrobial activity. Based on these results it can be assumed that *Lactarius* mushrooms could be a good source of natural antibiotics due to the high content of phenolic acids in their fruiting bodies.

Reviewing the available literature, the authors did not find any data on the inhibitory effect of the extracts of the tested mushrooms on the *S. aureus* biofilm. Therefore, our results can be considered the first report on biofilm inhibitory properties of these wild mushrooms. Biofilm represents a complex community of microorganisms that provides the microorganisms with protection from the effects of antibiotics, which is the most common cause of the occurrence of chronic infections and resistant strains [55]. Microorganisms in the biofilm are more resistant to antimicrobial agents than planktonic cells, therefore, treatment of biofilm with standard doses of commercially available antibiotics is often ineffective because it is known that to inhibit the growth and formation of biofilm, a dose up to 1000 times higher than the dose of antibiotics to inhibit the growth of planktonic cell is required [56]. Therefore, mushroom extracts, which are poorly investigated in terms of biofilm inhibition, represent a valuable resource in the search for new biologically active extracts/compounds that could inhibit biofilm formation better than antibiotics. Čuvalová et al. [57] investigated the potential of aqueous extracts of various mushrooms to inhibit the biofilm formation of *S. aureus*. They showed that the best activity was had by *A. mellea* (70.87%), followed by *P. ostreatus* (67%), *L. sulphureus* (64.14%) and *A. auricula-judae* (62.77%), while *M. procera* showed the lowest percentage of biofilm reduction (47.72%). On the other hand, *Grifola frondosa* extract demonstrated significant antibiofilm activity against clinical isolates of methicillin-resistant *S. aureus* (MRSA) [58]. Our results suggest that the antibiofilm activity of *Lactarius* species extracts should be considered an important approach in antimicrobial research. Therefore, further experiments are needed to evaluate which specific chemical compounds from the extracts of *Lactarius* species are responsible for their antibiofilm activity.

Considering that organic and phenolic acids are responsible for the antioxidant activity of mushroom extracts, the results of the chemical characterization of the tested species (Table 2 and Table 3) were in agreement with the obtained TBARS values. Studies by other authors showed that the methanol extract of the basidiocarp of *L. piperatus* from India exhibited moderate antioxidant activity (DPPH IC_50_ 1.57 mg/mL) [11], while the acetone extract of a sample from Serbia showed better activity (DPPH IC_50_ 0.033 mg/mL) [17]. The phenolic and polysaccharide fractions of the *L. vellereus* sample originating from Portugal showed weaker antioxidant activity (TBARS EC_50_ was 3.12 and 1.21 mg/mL, respectively) [10] compared to the activity of the ME and EE obtained in this study (TBARS EC_50_ was 0.32 and 0.30 mg/mL, respectively). The statements of numerous authors indicate a strong antioxidant activity of the genus *Lactarius* (TBARS 0.57–1.21 mg/mL) [6,10].

Literature data about the cytotoxicity of the *Lactarius* species is scarce. The acetone extract of *L. piperatus* showed very good cytotoxic activity (IC_50_ 37.83–65.94 μg/mL) on several different cell lines (HeLa, LS174, A549) [17], while ME *L. turpis* from Portugal showed no cytotoxic activity on tested healthy liver cells (PLP2) [6]. Thanks to intensive studies, around 30 types of mushrooms are now used as supplements due to their high antitumor activity [54]. From some mushrooms, active compounds (lentinan, krestin, psilocybin and schizophilan) have been isolated. They achieve antitumor activity through different mechanisms: induction of programmed cell death, immunomodulation, antioxidant potential, as well as by inhibiting proliferation and inflammation. It is well known that the mushroom compounds are highly effective when used alone in therapy, but also in combination with other treatment methods (surgical treatment, chemotherapy and radiation therapy) [59].

## 5. Conclusions

This study is the first report on the chemical composition, antibacterial and antibiofilm activity of three *Lactarius* species originating from Serbia. The research shows that the studied mushrooms represent a rich source of bioactive molecules with potent in vitro activities, especially in terms of antibiofilm activity. Based on the presented results, it can be concluded that the tested species show good potential for pharmaceutical application, but it is necessary to confirm the results obtained in vitro with in vivo tests and to investigate different mechanisms of action involved with specific bioactive compounds present in these species.

## Figures and Tables

**Table 1:** Identified *Lactarius* species.

Scientific Name	Code Name	Locality	Geographical Coordinates	Collection Year
*Lactarius piperatus* (L.) Pers.	Lp-061-2017	Kosmaj	44°28′22″ N 20°34′25″ E	2017
*Lactarius quietus* (Fr.) Fr.	Lq-071-2014	Divčibare	44°06′52″ N 20°02′15″ E	2014
*Lactarius vellereus* (Fr.) Fr.	Lv-082-2016	Kopaonik	43°17′30″ N 20°49′06″ E	2016

**Table 2:** Free sugars and organic acids composition of the studied mushrooms (mean ± SD, n = 3).

Constituent	*Lactarius piperatus*	*Lactarius quietus*	*Lactarius vellereus*
Free Sugars (g/100 g dw)			
Fructose	n.d.	n.d.	n.d.
Mannitol	5.8 ± 0.3 ^b^	3.67 ± 0.04 ^c^	13.47 ± 0.04 ^a^
Trehalose	17.8 ± 0.3 ^a^	0.34 ± 0.01 ^c^	0.77 ± 0.01 ^b^
**Total sugars**	**23.59 ± 0.04 ^a^**	**4.01 ± 0.03 ^c^**	**14.2 ± 0.4 ^b^**
Organic Acids (g/100 g dw)			
Oxalic acid	1.9 ± 0.2 ^c^	3.7 ± 0.1 ^b^	3.9 ± 0.2 ^a^
Quinic acid	0.3 ± 0.1	n.d.	n.d.
Malic acid	6 ± 1	n.d.	n.d.
Citric acid *	0.5 ± 0.1	n.d.	8.5 ± 3
Fumaric acid	0.33 ± 0.03	tr.	tr.
Shikimic acid	n.d.	n.d.	0.49 ± 0.02
**Total organic acids**	**9 ± 2 ^b^**	**3.7 ± 0.1 ^c^**	**12.4 ± 0.4 ^a^**

tr—traces; nd—not detected; Means with different letters differ significantly (*p* < 0.05). * Significant differences (*p* < 0.001) between samples were assessed by a Student’s *t*-test.

**Table 3:** Main fatty acids and tocopherols composition of the studied mushrooms (mean ± SD, n = 3).

	*Lactarius piperatus*	*Lactarius quietus*	*Lactarius vellereus*
Fatty acids (%)			
C16:0	4.93 ± 0.03 ^c^	20.1 ± 0.4 ^a^	10.0 ± 0.2 ^b^
C18:0	42.1 ± 0.2 ^b^	10.1 ± 0.1 ^c^	58.2 ± 0.9 ^a^
C18:1n9c	36.9 ± 0.4 ^b^	40.4 ± 0.4 ^a^	17.6 ± 0.8 ^c^
C18:2n6c	11.9 ± 0.2 ^c^	19.2 ± 0.1 ^a^	12.15 ± 0.02 ^b,c^
**Total SFA** (% of total FA)	**50.8 ± 0.3 ^b^**	**38.3 ± 0.5 ^c^**	**69.8 ± 0.8 ^a^**
**Total MUFA** (% of total FA)	**37.2 ± 0.4 ^b^**	**42 ± 1 ^a^**	**17.9 ± 0.9 ^c^**
**Total PUFA** (% of total FA)	**12.0 ± 0.2 ^b^**	**19.8 ± 0.1 ^a^**	**12.31 ± 0.02 ^b^**
Tocopherols (µg/100 g dw)			
α-Tocopherol	4 ± 1	n.d.	n.d.
β-Tocopherol	47 ± 2 ^c^	69.0 ± 0.8 ^b^	1391 ± 21 ^a^
γ-Tocopherol	26.1 ± 0.1	n.d.	n.d.
δ-Tocopherol	3.1 ± 0.7	n.d.	n.d.
**Total Tocopherols**	**80 ± 3 ^b^**	**69.0 ± 0.8 ^c^**	**1391 ± 21 ^a^**

n.d.—not detected; Means with different letters differ significantly (*p* < 0.05).

**Table 4:** Phenolic and related compounds of the extracts of the studied mushrooms (mean ± SD, n = 3).

Phenolic and Related Compounds (µg/g ext)	LP-ME	LP-EE	LQ-ME	LQ-EE	LV-ME	LV-EE
*p*-Hydroxybenzoic acid	n.d.	n.d.	795 ± 3 ^b^	4735 ± 9 ^a^	2.0 ± 0.2 ^d^	2.5 ± 0.2 ^c^
Protocatechuic acid *	n.d.	n.d.	n.d.	n.d.	4.2 ± 0.3	11.4 ± 0.5
**Total phenolic compounds**	**-**	**-**	**795 ± 3 ^b^**	**4735 ± 9 ^a^**	**6.2 ± 0.5 ^d^**	**13.9 ± 0.40 ^c^**
Cinnamic acid	8.5 ± 0.4 ^c^	5.2 ± 0.2 ^d^	23.8 ± 0.1 ^b^	126 ± 3 ^a^	n.d.	n.d.

n.d.—not detected; LP-ME—*L. piperatus* methanolic extract, LP-EE—*L. piperatus* ethanolic extract, LQ-ME—*L. quietus* methanolic extract, LQ-EE—*L. vellereus* ethanolic extract LV-ME—*L. vellereus* methanolic extract, LV-EE—*L. vellereus* ethanolic extract. Means with different letters differ significantly (*p* < 0.05); * Significant differences (*p* < 0.001) between samples were assessed by a Student’s *t*-test.

**Table 5:** Antibacterial activity of methanolic and ethanolic extracts of tested *Lactarius* species (mg/mL).

		LP-ME	LP-EE	LQ-ME	LQ-EE	LV-ME	LV-EE	Amoxicillin with Clavulanic Acid	Cefixime
*Micrococcus* *luteus*	MIC	0.8	6.25	3.1	3.1	1.5	1.5	0.0002	0.002
	MBC	1.6	12.5	6.2	6.25	3.1	3.1	0.0004	0.003
*Rothia mucilagenosa*	MIC	0.8	3.1	6.25	0.6	1.6	0.8	0.007	0.002
	MBC	1.6	6.25	12.5	3.1	3.1	1.6	0.014	0.003
*Streptococcus agalactiae*	MIC	0.4	3.1	0.2	0.6	0.8	0.8	0.007	0.002
	MBC	0.8	6.25	0.4	3.1	1.6	1.6	0.014	0.004
*Streptococcus anginosus*	MIC	12.5	3.1	3.1	3.1	0.8	0.8	0.028	0.0002
	MBC	25	6.25	6.25	6.2	1.6	1.6	0.056	0.0004
*Streptococcus constellatus*	MIC	0.8	6.25	1.56	3.1	1.56	1.56	0.0002	0.0002
	MBC	1.56	12.5	3.1	6.2	3.1	3.1	0.0004	0.0004
*Streptococcus dysgalactiae*	MIC	0.4	1.5	1.5	0.8	1.5	1.5	0.007	0.0002
	MBC	0.8	3.1	3.1	1.6	3.1	3.1	0.014	0.0004
*Streptococcus oralis*	MIC	6.2	3.1	0.8	3.1	6.25	3.1	0.0004	0.002
	MBC	12.5	6.25	1.6	6.2	12.5	6.25	0.001	0.004
*Streptococcus parasanquinis*	MIC	0.4	3.1	0.2	0.6	0.8	0.8	0.004	0.003
	MBC	0.8	6.25	0.4	3.1	1.6	1.6	0.01	0.006
*Streptococcus pseudopneumoniae*	MIC	0.8	3.1	1.6	6.2	6.25	1.6	0.001	0.013
	MBC	1.6	6.25	3.1	12.5	12.5	3.1	0.002	0.027
*Streptococcus pyogenes*	MIC	3.1	0.8	0.4	6.25	3.1	0.8	0.0004	0.0008
	MBC	6.2	1.6	0.8	12.5	6.25	1.6	0.001	0.002
*Streptococcus salivarius*	MIC	1.6	1.6	0.4	3.1	6.25	3.1	0.01	0.013
	MBC	3.1	3.1	0.8	6.2	12.5	6.25	0.014	0.027
*Staphylococcus aureus*	MIC	12.5	25	12.5	12.5	25	12.5	0.001	0.003
	MBC	25	50	25	25	30	25	0.002	0.006
*Staphylococcus hominis*	MIC	15	15	3.75	3.75	7.5	7.5	0.004	0.002
	MBC	30	30	7.5	7.5	15	15	0.007	0.004
*Staphylococcus warnerii*	MIC	6.25	6.25	6.25	3.1	6.25	12.5	0.001	0.003
	MBC	12.5	12.5	12.5	6.25	12.5	25	0.002	0.006
*Enterobacter cloacae*	MIC	12.5	25	6.25	3.1	6.25	1.6	0.028	0.003
	MBC	25	50	12.5	6.2	12.5	3.1	0.056	0.007
*Stenotrophomonas maltophilia*	MIC	25	25	6.25	25	25	12.5	0.003	0.003
	MBC	50	50	12.5	50	50	25	0.007	0.006

MIC—minimal inhibitory concentration; MBC—minimal bactericidal concentration. LP-ME—*L. piperatus* methanolic extract, LP-EE—*L. piperatus* ethanolic extract, LQ-ME—*L. quietus* methanolic extract, LQ-EE—*L. vellereus* ethanolic extract LV-ME—*L. vellereus* methanolic extract, LV-EE—*L. vellereus* ethanolic extract.

**Table 6:** Antioxidant and cytotoxic activities of the different *Lactarius* extracts (GI_50_ (μg/mL) ± SD).

Type of Extract	Antioxidant Activity (EC_50_, mg/mL) *	Cytotoxicity to Non-Tumor Cell Lines (GI_50_, μg/mL) **	Cytotoxicity to Tumor Cell Lines (GI_50_, μg/mL) **			
	TBARS	PLP2	HeLa	HepG2	MCF-7	NCI-H460
		(porcine liver primary culture) ***	(cervical carcinoma)	(hepatocellular carcinoma)	(breast carcinoma)	(non-small cell lung cancer)
LP-ME	0.41 ± 0.08 ^b^	>400	229 ± 10 ^c^	263 ± 6 ^c^	216 ± 11 ^b^	>400
LP-EE	1.39 ± 0.06 ^a^	>400	266 ± 17 ^a^	>400	276 ± 11 ^a^	>400
LQ-ME	0.17 ± 0.01 ^e^	347 ± 12	196 ± 3 ^d^	191 ± 14 ^d^	202 ± 8 ^c^	220 ± 6 ^b^
LQ-EE	0.23 ± 0.02 ^d^	>400	>400	312 ± 20 ^a^	>400	>400
LV-ME	0.32 ± 0.01 ^c^	>400	253 ± 8 ^b^	271 ± 3 ^b^	>400	331 ± 3 ^a^
LV-EE	0.30 ± 0.01 ^c^	163 ± 7	71 ± 3 ^e^	80 ± 6 ^e^	87 ± 4 ^d^	83 ± 3 ^c^

EC50: extract concentration corresponding to 50% of antioxidant activity (TBARS); * Trolox EC_50_ values: 5.4 ± 0.3 μg/mL (TBARS); GI_50_ values correspond to the sample concentration responsible for 50% inhibition of growth in a primary culture of liver cells-PLP2 or human tumor cell lines; ** Ellipticine GI_50_ values: 1.44 ± 0.08 μg/mL (PLP2), 0.86 ± 0.04 μg/mL (HeLa), 1.12 ± 0.06 μg/mL (HepG2), 0.91 ± 0.04 μg/mL (MCF-7) and 1.0 ± 0.1 μg/mL (NCI-H460); *** Significant differences (*p* < 0.001) between the two extracts were assessed by a Student’s *t*-test. LP-ME—*L. piperatus* methanolic extract, LP-EE—*L. piperatus* ethanolic extract, LQ-ME—*L. quietus* methanolic extract, LQ-EE—*L. vellereus* ethanolic extract LV-ME—*L. vellereus* methanolic extract, LV-EE—*L. vellereus* ethanolic extract; Means with different letters differ significantly (*p* < 0.05).

## Data Availability

Not applicable.
